# Factors Associated with Acute Telemental Health Consultations in Older Veterans

**DOI:** 10.5811/westjem.17996

**Published:** 2024-04-09

**Authors:** Erica C. Koch, Michael J. Ward, Alvin D. Jeffery, Thomas J. Reese, Chad Dorn, Shannon Pugh, Melissa Rubenstein, Jo Ellen Wilson, Corey Campbell, Jin H. Han

**Affiliations:** * Vanderbilt University Medical Center, Department of Emergency Medicine, Nashville, Tennessee; † Tennessee Valley Healthcare System, Geriatric Research, Education, and Clinical Center, Nashville, Tennessee; ‡ Vanderbilt University School of Nursing, Nashville, Tennessee; § Vanderbilt University Medical Center, Department of Biomedical Informatics, Nashville, Tennessee; ∥ Tennessee Valley Healthcare System, Nursing Services, Nashville, Tennessee; ¶ Vanderbilt University Medical Center, Department of Psychiatry, Nashville, Tennessee; # Tennessee Valley Healthcare System, Psychiatric Services, Nashville, Tennessee; ** Veterans Affairs Quality Scholars Program, Nashville, Tennessee

## Abstract

**Introduction:**

The United States Veterans Health Administration is a leader in the use of telemental health (TMH) to enhance access to mental healthcare amidst a nationwide shortage of mental health professionals. The Tennessee Valley Veterans Affairs (VA) Health System piloted TMH in its emergency department (ED) and urgent care clinic (UCC) in 2019, with full 24/7 availability beginning March 1, 2020. Following implementation, preliminary data demonstrated that veterans ≥65 years old were less likely to receive TMH than younger patients. We sought to examine factors associated with older veterans receiving TMH consultations in acute, unscheduled, outpatient settings to identify limitations in the current process.

**Methods:**

This was a retrospective cohort study conducted within the Tennessee Valley VA Health System. We included veterans ≥55 years who received a mental health consultation in the ED or UCC from April 1, 2020–September 30, 2022. Telemental health was administered by a mental health clinician (attending physician, resident physician, nurse practitioner, or physician assistant) via iPad, whereas in-person evaluations were performed in the ED. We examined the influence of patient demographics, visit timing, chief complaint, and psychiatric history on TMH, using multivariable logistic regression.

**Results:**

Of the 254 patients included in this analysis, 177 (69.7%) received TMH. Veterans with high-risk chief complaints (suicidal ideation, homicidal ideation, or agitation) were less likely to receive TMH consultation (adjusted odds ratio [AOR]: 0.47, 95% confidence interval [CI] 0.24–0.95). Compared to attending physicians, nurse practitioners and physician assistants were associated with increased TMH use (AOR 4.81, 95% CI 2.04–11.36), whereas consultation by resident physicians was associated with decreased TMH use (AOR 0.04, 95% CI 0.00–0.59). The UCC used TMH for all but one encounter. Patient characteristics including their visit timing, gender, additional medical complaints, comorbidity burden, and number of psychoactive medications did not influence use of TMH.

**Conclusion:**

High-risk chief complaints, location, and type of mental health clinician may be key determinants of telemental health use in older adults. This may help expand mental healthcare access to areas with a shortage of mental health professionals and prevent potentially avoidable transfers in low-acuity situations. Further studies and interventions may optimize TMH for older patients to ensure safe, equitable mental health care.

Population Health Research Capsule What do we already know about this issue?

*There is a widespread shortage of mental health professionals in the US, which decreases access to timely emergency mental healthcare.*

What was the research question?

*What factors are associated with older veterans receiving acute, unscheduled telemental health (TMH) vs in-person consults?*

What was the major finding of the study?

*High-risk chief complaints (suicidal or homicidal ideation, or agitation) were associated with decreased TMH use (OR 0.39, 95% CI 0.18–0.81). Type of clinician and location of care were also associated with TMH use.*

How does this improve population health?

*TMH represents an opportunity to expand access to mental healthcare, thereby reducing potentially unnecessary patient transfers and shortening boarding times*.

## INTRODUCTION

In 2020, 52.9 million people in the United States (US) suffered from a mental health or substance use disorder.^1^^,^^2^ Emergency department (ED) visits and admissions for psychiatric concerns continue to increase.^3^^–^^7^ Despite the increased demand, there is a widespread mental health professionals shortage in the US, which negatively affects access to timely, efficient mental healthcare for society’s most vulnerable populations. An estimated 7,632 clinicians are needed to bridge the gap in low-resourced areas.^8^ Approximately 66% of rural or partially rural counties are designated by the federal government as mental health professional shortage areas.^8^ Patients in these areas have been found to have worse health outcomes, including shorter life expectancy and higher rate of suicide.^9^^–^^11^ Innovative solutions are needed to address these key gaps to expand access to equitable mental health services, particularly in the setting of acute crises.

Telehealth was first described in clinical practice in the late 1950s.^12^ Over the past two decades, use has expanded in a variety of clinical settings.^13^ The Veterans Health Administration has adopted telehealth across a variety of settings, including mental health complaints.^14^ By 2016, nearly half of EDs in the US reported the use of telehealth, with 20% using it for mental health purposes (telemental health [TMH]).^15^^,^^16^ The use of TMH in routine ED clinical practice grew dramatically during the COVID-19 pandemic.^5^ For many EDs, it is the only avenue to emergency psychiatric care.^15^

On March 1, 2020, the Tennessee Valley Veterans Affairs Health System implemented full-time TMH for veterans who presented to the ED for mental health complaints. Both TMH and in-person consultations performed by a mental health clinician were available 7 days a week, 24 hours a day, including holidays, at the ED and during all operating hours at the UCC (daily 8 am – 8 pm). Consultation modality was left to the choice of the mental health clinician. In-person clinician coverage was always available by an attending physician, resident physician, nurse practitioner, or physician assistant during facility operating hours. Capabilities did not change depending on the role of the clinician. A more detailed description of the program is provided elsewhere.^17^ Despite the implementation of this TMH program, preliminary data showed 20% of mental health consultations still occurred in person.^17^ Veterans who received in-person mental health evaluations were notably older compared to those receiving TMH, with 31% in-person consults occurring in veterans ages ≥65 vs 18% of TMH consults.^17^

Older patients with mental health complaints face unique challenges in the emergency setting. Attention to these patients during the implementation of new processes of care is vital to ensure they receive high-quality mental health evaluation. With the exponential growth projected for the older population in the US, understanding factors associated with variability of TMH use will inform future implementation and sustainability in acute care settings.^18^ In this study we sought to examine factors associated with older veterans receiving TMH consultations in acute, unscheduled, outpatient settings to identify potential barriers to widespread use of TMH in the ED. Encounters involving patients older than 75, urban location, resident physicians, and higher acuity were hypothesized to be more likely to occur in person.

## METHODS

### Study Design, Setting, and Patient Population

This was an exploratory, retrospective, cohort study conducted at the Tennessee Valley VA Health System ED and urgent care clinic (UCC).^20^ Described in more detail elsewhere, this TMH program was initially piloted during limited hours in 2019 and then went live with 24/7 coverage in March 2020 with the onset of the COVID-19 pandemic.^17^ Patients were initially evaluated by an ED or UCC clinician (attending physician, resident physician, nurse practitioner, or physician assistant) and determined to need mental health consultation. A consult order was then requested through the electronic health record (EHR) with direct communication between the emergency physician and on-call mental health clinician (nurse practitioner, physician assistant, or attending psychiatrist). Consult modality was left to the decision of the on-call mental health clinician. The TMH visit was provided via Apple iPad (Apple Inc, Cupertino, CA) with audio and visual capabilities, whereas in-person evaluations were performed by the same mental health clinician in the ED or UCC. Both in-person and TMH consultations were available 24/7 in the ED and during operating hours of the UCC.

We included veterans who were ≥55 years and received a mental health consultation in the ED between April 1, 2020–September 30, 2022. Since there is no universally accepted age that defines “older age,” we chose 55 years old as the cut-off to maximize our sample size while maintaining a median age of 65 years old, a traditional cut-point. Non-veterans without service benefits, direct admissions who did not present through the ED, and patients with a missing modality of consultation were excluded. For veterans with multiple ED mental health encounters, only the first consultation encounter was included. Of 1,478 initial visits within the study period, we selected 510 charts to review; 497 had complete mental health consultations in the chart. A substantial proportion of patients received TMH during the study period. Therefore, 2–3 TMH consultations were included for each in-person consultation. We balanced the number of charts selected for each month of the study to reduce temporal bias. We then excluded all patients <55 years from this analysis. This study was approved by the local institutional review board as exempt.

### Data Collection

We designed the chart review methodology to follow accepted guidelines.^21^ Data was manually extracted from the VA EHR and Clinical Data Warehouse. The following patient factors were included in this analysis: age; race; gender; marital status; rurality; ED triage chief complaint; mental health history; total active number of psychoactive medications; and presence of additional non-psychiatric medical complaint (eg, chest pain). Rurality was determined by the Rural-Urban Commuting Area Codes based on the patient’s ZIP code.^22^ We considered the following system-level factors: location (ED vs UCC); timing of presentation (9 am – 5 pm or nights/weekends) and mental health clinician type (nurse practitioner, physician assistant, resident physician, or attending physician).

Patient demographics, visit date, homelessness, psychiatric history, and medications were manually abstracted by a physician (ECK) and nurse (SP). Senior authors trained abstractors prior to data collection. Each reviewer underwent mentored training on how to review each chart with a trial period of manual double-checking by the senior author to ensure competency. Each chart was reviewed by either the physician or nurse reviewer and then was carefully double-checked by the same reviewer for inaccuracies. Each chart was reviewed by one person. Data abstraction forms were used, and the data was compiled using REDCap electronic data capture tools hosted at US Department of Veterans Affairs.

We used the total number of psychiatric conditions documented in the EHR prior to the index ED visit to determine psychiatric comorbidity burden. Any mention of suicidal ideation, homicidal ideation, and agitation qualified as high-risk mental health chief complaints, regardless of whether this was the patient’s primary reason for ED evaluation. Additional medical reasons for the ED visit were collected by reviewing triage and physician documentation.

### Outcome Measures

The primary dependent variable of interest was receipt of TMH vs in-person mental health consultation by a mental health clinician who was an attending physician, resident physician, or nurse practitioner.

### Data Analysis

We reported central tendency and dispersion as medians and interquartile ranges for continuous variables. Categorical variables were reported as frequencies and percentages. A multivariable logistic regression analysis was performed to determine factors associated with use of TMH. We created a moderately saturated model with 7–8 covariates to minimize overfitting.^23^ Given the small sample size, independent variables were ranked a priori based on expert opinion from psychiatrists (EJW, CC) and emergency physicians (MJW, JHH) who routinely care for mental health patients. The top seven ranked factors for TMH vs in-person mental health evaluation included age, race, high-risk chief complaint, presence of dementia, urban location, timing of presentation, and history of substance abuse. To explore additional factors associated with TMH vs in-person mental health consultation, we performed a highly saturated model incorporating all factors into the multivariable logistic regression model. Because site (ED vs UCC) of patient presentation may have strongly influenced TMH vs in-person mental health, this factor was incorporated into the models. Adjusted odds ratios (aOR) with 95% confidence intervals (CI) are reported. We conducted all statistical analyses with R statistical software, v3.6.2 (The R Project for Statistical Computing, Vienna, Austria).

## RESULTS

Of the 510 health records reviewed, 254 patients met age inclusion criteria (≥55 years of age) and were included in the study. Characteristics of this older cohort vs the entire cohort of charts reviewed is included as a supplemental table. Of those eligible, 177 (69.7%) veterans received TMH consultations, and 77 (30.3%) veterans received an in-person evaluation. There were no missing data points on chart review. In the unadjusted results, UCC location and consultation performed by nurse practitioners and physician assistants was associated with a statistically significant trend towards TMH use (Table 1). Consultations performed by resident mental health physicians were more likely to occur in person but represented few consults overall (Table 1). Age, race, presence of dementia or substance use disorder in medical history, total psychoactive medications, psychiatric comorbidity burden, homelessness, and marital status were not associated with significant differences in consult modality.

**Table 1.** Baseline demographic data of patients presenting to the emergency department or urgent care center receiving psychiatric consultation.

Variable	In-person (n = 77)	Telemental health (n = 177)
Age, (years)	65 [61, 71]	65 [61, 70]
Gender, n (%)		
Female	3 (3.9)	14 (7.9)
Male	74 (96.1)	163 (92.1)
Race, n (%)		
Black	30 (39.0)	72 (40.7)
Non-Black	47 (61.0)	105 (59.3)
Marital status, n (%)		
Married	26 (33.8)	44 (24.9)
Unmarried/unknown	51 (66.2)	133 (75.1)
Chief complaint risk, n (%)		
Low	49 (63.6)	130 (73.4)
High	28 (36.4)	47 (26.6)
History of dementia, n (%)		
Yes	10 (13.0)	18 (10.2)
No	67 (87.0)	159 (89.8)
Location, n (%)		
ED	76 (98.7)	151 (85.3)
UCC	1 (1.3)	26 (14.7)
Rural, n (%)		
Rural	24 (31.2)	45 (25.4)
Urban	53 (68.8)	132 (74.6)
ESI score ≥ 2, n (%)	77 (100.0)	177 (100.0)
ESI score, n (%)		
<3	22 (28.6)	61 (34.5)
≥3	55 (71.4)	116 (65.5)
Timing of presentation, n (%)		
Off hours	28 (36.4)	64 (36.2)
Business hours	49 (63.6)	113 (63.8)
History of substance abuse, n (%)		
No	36 (46.8)	74 (41.8)
Yes	41 (53.2)	103 (58.2)
Mental health clinician type, n (%)		
Attending physician	62 (80.5)	123 (69.5)
Resident physician	7 (9.1)	1 (0.6)
Nurse practitioner or physician assistant	8 (10.4)	53 (29.9)
Total psychoactive medications, median [IQR]	2.00 [1.00, 4.00]	2.00 [1.00, 4.00]
Total psychiatric comorbidities, median [IQR]	1.00 [1.00, 2.00]	2.00 [1.00, 2.00]
Additional triage medical complaint, n (%)		
No	48 (62.3)	118 (66.7)
Yes	29 (37.7)	59 (33.3)
Homelessness, n (%)		
No	64 (83.1)	144 (81.4)
Yes	13 (16.9)	33 (18.6)
CCI score, median [IQR]	2.00 [1.00, 4.00]	2.00 [1.00, 5.00]

*ESI*, Emergency Severity Index; *IQR*, interquartile range; *CCI*, Charlson Comorbidity Index; *UCC*, urgent care clinic; *ED*, emergency department.

We then performed multivariable logistic regression analysis. Models were adjusted for location to account for site practice differences at the ED and UCC, as the UCC performed nearly all consults via TMH. Table 2 demonstrates a moderately saturated risk model. No factors were significantly associated with TMH use beyond urgent care location (AOR 15.15, 95% CI 1.98–116.04). In a highly saturated model, patients evaluated by resident physicians were less likely to receive TMH (AOR 0.04, 95% CI: 0.00–0.58), while those evaluated by nurse practitioners and physician assistants received it more frequently (A5.07, 95% CI: 2.13–12.03), compared to attending physicians (Table 3). Patients with high-risk chief complaints (suicidal ideation, homicidal ideation, or agitation) were less likely to receive TMH (AOR: 0.39, 95% CI: 0.18–0.81) in the highly saturated risk model (Table 3). Gender, age, race, comorbidity burden, timing of presentation, history of substance use disorder, history of dementia, and homelessness were not associated significant differences in consult modality.

**Table 2.** Multivariable regression analysis – moderately saturated model.

Variable	Adjusted odds ratio	95% confidence interval
Age	1.02	0.98–1.07
Race–Non-black	0.87	0.35–2.13
High-risk chief complaint	0.54	0.29–1.00
History of dementia	0.86	0.35–2.13
Location at UCC	15.15	1.98–116.04
Urban location	1.54	0.82–2.88
Timing of presentation during off hours	1.16	0.64–2.09
History of substance abuse	1.33	0.72–2.44

*UCC*, urgent care clinic.

**Table 3.** Multivariable regression analysis – highly saturated model.

Variable	Odds ratio	95% confidence interval
Age	1.04	0.99–1.09
Gender–male	0.35	0.07–1.70
Race–non-Black	0.79	0.40–1.57
Marital status–unmarried or unknown	1.11	0.54–2.30
High-risk chief complaint	0.39	0.18–0.81
History of dementia	0.51	0.18–1.42
UCC location	29.11	2.76–306.99
Urban location	1.48	0.74–2.98
Timing of presentation during off hours	1.36	0.70–2.63
History of substance abuse	1.14	0.57–2.26
Mental health clinician type		
Nurse practitioner or physician assistant	5.07	2.13–12.03
Resident physician	0.04	0.00–0.58
Total psychoactive medications	1.11	0.94–1.32
Total psychiatric comorbidities	1.18	0.90–1.54
Additional triage medical complaint	0.72	0.37–1.40
Homelessness	1.13	0.47–2.71
CCI score	1.09	0.97–1.23

*UCC*, urgent care clinic; *CCI*, Charlson Comorbidity Index.

## DISCUSSION

In an older cohort of veterans presenting to the ED or UCC with acute psychiatric complaints, we found that high-risk psychiatric chief complaints, clinician type, and location of the mental health consult were key drivers of consultation modality. Specifically, we observed that patients with high-risk psychiatric chief complaints (suicidal ideation, homicidal ideation, and agitation) were more likely to receive in-person consultations. Resident physicians performing consults were less likely to use TMH, while nurse practitioners and physician assistants were more likely to choose TMH. The UCC used TMH near universally.

The moderately saturated risk model of most highly ranked a priori factors showed AORs greater than 1 in urban location, timing of presentation during off hours, and history of substance use disorder. However, the 95% CI were too wide to be significant. These findings were similar in the highly saturated model. While not statistically significant, these factors may hold clinical relevance. Further studies with a higher sample size are needed to clarify any significance.

One potential explanation for reduced use among higher severity complaints is that mental health clinicians may feel more compelled to conduct in-person consultation in higher acuity situations because this is what they are most familiar with. Practice changes such as the use of TMH may create a disruption as physicians struggle to “unlearn” what they are most familiar with prior to establishing a new practice pattern.^24^ Alternatively, as recognized by the Society for Academic Emergency Medicine Consensus Conference on Emergency Telehealth, little research has been done on the quality and safety of telehealth.^25^ Recent work has sought to address this. Evidence suggests patients presenting with acute psychosis may tolerate telehealth well.^26^^,^^27^ Telemental health has been found to have no difference in long-term outcomes of rehospitalization and death in patients with suicidal ideation and suicide attempts compared to in-person consultation.^26^^,^^28^ Additionally, recent work has suggested that TMH is not associated with increased 30-day return visits, readmissions, or death compared to in-person evaluations in acute care settings.^26^ Therefore, ED and mental health clinicians should be educated on the safety of TMH in older ED patients with high-risk mental health chief complaints.

Prior research demonstrated that clinicians contribute substantial variability to the decision to use telehealth and may partially explain why there are such differences in the use of TMH by clinician type (ie, resident physicians vs nurse practitioners and physician assistants).^29^ There were no differences in clinician scheduling that could account for the findings in our study. All mental health clinicians, including residents, were available to perform in-person or TMH evaluations. Therefore, location did not make residents more or less likely to evaluate patients in person. The pandemic demonstrated variability in telehealth use with clinician factors having a greater influence on the use of video telehealth when compared with patient factors.^29^ Moreover, prior studies indicate there are variabilities in patients who are offered telehealth despite being video-capable.^30^ Prior qualitative studies suggest that increased exposure to telehealth improved clinician attitudes, while perceptions of complexity within the process led to reduced utilization.^31^ Further research is needed to better understand whether inequities and any contributing factors exist.

Systems with unanimous leadership buy-in and policies use telehealth more frequently.^31^ Despite the availability of an in-person mental health clinician, the UCC used TMH for nearly every encounter. It is plausible that similar systemic factors may be contributing to this phenomenon. Investigating the policies and decision-making processes through qualitative studies could shed light on the underlying reasons for the near-universal use of TMH at the UCC, as factors not captured in this study are likely involved.

Reluctance to adopt TMH may contribute to potentially avoidable transfers in EDs with limited mental health resources. Prior research found that mental health patients were the most likely to be transferred from VA EDs and represent the largest group of potentially avoidable transfers, defined as those transfers rapidly discharged from the ED or within 24 hours from hospital admission (without a procedure).^32^ Our findings suggest that mental health clinicians felt comfortable evaluating patients via TMH in low-acuity situations. In places without access to in-person mental health consultation, patients with lower acuity complaints may be evaluated and safely discharged via TMH, reducing the risk of unnecessary transfer.^33^

We identified only one resident TMH encounter throughout the entire study period. As residents generally rotate between multiple VA and non-VA clinical services, this finding may be due to lack of familiarity with the process in this system. Due to the low overall number of consultations performed by residents, it is difficult to draw conclusions regarding this data. Educational initiatives targeting telehealth use among resident physicians may increase familiarity with TMH.^34^^,^^35^ As telehealth expanded across multiple specialties during the pandemic, medical training curricula could be adapted to include telehealth initiatives.^34^^,^^35^

## LIMITATIONS

Limitations of this study included a small sample size. Our sample size may have been too small to identify risk factors for TMH use. Additionally, because this study was conducted in a single center it may not be generalizable to other settings. Risk factors identified in our exploratory analysis and the significant associations observed may have been secondary to overfitting as statistical significance was only noted in the highly saturated model. As a result, these findings should be confirmed in a larger sample size. Additionally, the VA has a low proportion of women veterans (estimated 11.5%), potentially limiting the generalizability of our study outside the VA population.^36^ Further studies outside the VA population are needed to assess for any gender-specific differences that may impact consult modality choice.

The ED/UCC clinician and mental health clinician generally had a verbal conversation on call prior to mental health consultation. These conversations may have influenced modality choice by the mental health clinician. Our quantitative data would not have been able to capture these conversations. Further qualitative work may bridge this gap to understand a clinician’s modality choice.

There are potential confounders to this study that were not accounted for. Severity of illness likely affects both the likelihood of acute care presentation and the consult modality choice. While we adjusted for high-risk psychiatric complaints to account for severity of illness, residual confounding likely still exists. Encounters that occurred during the COVID-19 pandemic also likely influenced both the likelihood of acute care presentation and the consult modality choice. More mental health clinicians may have opted for TMH to reduce the risk of virus transmission, especially during periods of widespread COVID-19 transmission. Patients may have also been more fearful of presenting to the ED for care during these times. Ongoing post-pandemic data analysis both at this facility and externally should be performed to evaluate the effects of the COVID-19 pandemic on TMH use.

## CONCLUSION

In this exploratory retrospective analysis, illness severity, location, and clinician characteristics appeared to influence use of telemental health in patients over age 55. Lower acuity, older patients represent a patient population with whom more clinicians would be comfortable using TMH. For resource-poor settings, TMH may represent an opportunity to expand access to mental healthcare in shortage areas and reduce potentially unnecessary patient transfers that could otherwise be prevented via remote consultation. Further research is needed to examine hesitancy to adopt TMH in more acutely ill populations and the generalizability of the findings presented in this work.
